# Female and functional urology in 2010

**DOI:** 10.4103/0970-1591.65391

**Published:** 2010

**Authors:** Andrew Thorpe

**Affiliations:** Consultant Urological Surgeon, Department of Urology, The Freeman Hospital, Newcastle upon Tyne, NE7 7DN, UK. E-mail: Andrew.thorpe@nuth.nhs.uk

It was with great pleasure that I accepted the offer from Professor Kekre to guest-edit his seminar edition on Female Urology, Incontinence and Bladder Dysfunction for the Indian Journal of Urology. Functional Urology has made huge strides in the last decade, in the management and treatment of patients with stress incontinence, overactive bladder, and in neurological patients who suffer from urological symptoms. There has also been an increasing interest not only in the United Kingdom, but also in Europe and North America, of urologists in treating women with prolapse.

I am sure that this supplement will give quite a comprehensive overview to any trainee who is currently preparing to take higher examinations in urology, and to all consultant urologists requiring an update in the arena of functional urology. I would like to thank all of my colleagues from the UK who agreed to write review articles for this supplement, they are all recognized as national, and in a lot of cases, international experts in their area.

To start the seminar, we have included a paper on the urodynamic assessment of patients with symptoms of stress urinary incontinence (SUI). Getting this right is of paramount importance; it does seem quite obvious and logical that unless the correct diagnosis is made, the correct remedial treatment cannot be given, but it is surprising that going into the 21^st^ century, a huge number of units worldwide still practice urodynamics poorly, and have poor quality control of their charts in place – if we cannot get this right then we are on a slippery slope before we have even started contemplating treatment.

The overactive bladder causes significant morbidity to patients, and is a source of a huge financial burden. We have two excellent papers in this issue of the journal; the first is concerned with the medical management of the overactive bladder (OAB), and gives us a very comprehensive overview of the treatment options. The second paper on OAB outlines the options that are available for that often perplexing group of patients who have failed medical management, and return needing more invasive therapy. The authors have given a very comprehensive overview of the options available, which range from intra-vesical botulinum toxin injections, through sacral neuromodulation, to the use of clam enterocystoplasty, which despite the newer innovations in treatment, still remains a very effective operative option. SUI still remains a huge problem on a worldwide basis. Not enough women are coming forward with this problem – it still remains a huge taboo, and many women still accept stress incontinence as part of the aging process. A further problem is one of education, in terms of what is now on offer for this condition, not only to the women themselves but also to their primary care practitioners. This edition of the journal provides two reviews which complement each other very well. The first is an overview on the operative alternatives that are now available in the management of women with SUI, the second review explains the complications that may occur when biomaterials are used for this type of surgery, and how they are best corrected. Female urinary retention is rare, and may be multi-factorial. We have a very comprehensive review in this supplement, which fully explains the causes and best management strategies in women presenting with this particular problem.

Finally, we have three papers which cover more sub-specialist areas within the female urology and bladder dysfunction remit. Most urologists will, in their day-to-day working lives, treat patients who have neurological problems occurring secondary to neurological disorders. In the UK, the treatment of patients with spinal injuries has been centralized into spinal injuries’ units. All patients in these units, whether as in-patients or outpatients, have access to a urologist to look after both upper and lower tract problems. The review article in this supplement covers the issues facing spinal-injured patients with urological problems admirably, and will be extremely helpful to all the readers who have an interest in this often very difficult area. Traditionally, prolapse repair has been a service offered by the urogynecologists, but within the UK and elsewhere, there has been a move to bring this area into the remit of reconstructive urologists who have an interest in female urology. The article written for this seminar issue comes from a urological centre where just such a service is offered, and provides a comprehensive insight into the management of both simple and complex vesico-vaginal fistulae.

Our final paper covers the topic of post-prostatectomy incontinence. We are now well into the prostate-specific antigen (PSA) and transrectal ultrasound era. This has generated a huge number of cases of early prostate cancer, with a significant percentage of patients electing to undergo radical retropubic prostatectomy (whether it is open, laparoscopic or robotically-assisted). This in turn generates a number of cases of post-prostatectomy incontinence, whatever the original operative route, ranging from mild through to quite severe urinary incontinence, which may have profound socio-psychological effects on individuals. The paper reviews and discusses the range of options that are available for treating this problem.

I do hope you enjoy reading this supplement, it is wide-ranging, and I hope thought-provoking. To any urological practitioner who has an interest in functional urology it will provide a timely update.

